# Mitigation of drought stress in chili plants (*Capsicum annuum* L.) using mango fruit waste biochar, fulvic acid and cobalt

**DOI:** 10.1038/s41598-024-65082-5

**Published:** 2024-06-20

**Authors:** Misbah Hareem, Subhan Danish, Sami Al Obaid, Mohammad Javed Ansari, Rahul Datta

**Affiliations:** 1 Department of Environmental Sciences, Woman University Multan, Multan, Punjab Pakistan; 2 Pesticide Quality Control Laboratory, Agriculture Complex, Old Shujabad Road, Multan, Punjab Pakistan; 3 Department of Botany and Microbiology, College of Science, King Saud University, PO Box 2455, 11451 Riyadh, Saudi Arabia; 4 Department of Botany, Hindu College Moradabad (Mahatma Jyotiba Phule Rohilkhand University Bareilly), Moradabad, India; 5 Department of Geology and Pedology, Faculty of Forestry and Wood Technology, Mendel University in Brno, Zemedelska 1, 61300 Brno, Czech Republic

**Keywords:** Activated carbon, Antioxidants, Chlorophyll content, Growth attributes, Nutrients concentration, Organic amendment, Osmotic stress, Plant sciences, Plant stress responses, Abiotic, Drought

## Abstract

Drought stress can have negative impacts on crop productivity. It triggers the accumulation of reactive oxygen species, which causes oxidative stress. Limited water and nutrient uptake under drought stress also decreases plant growth. Using cobalt and fulvic acid with biochar in such scenarios can effectively promote plant growth. Cobalt (Co) is a component of various enzymes and co-enzymes. It can increase the concentration of flavonoids, total phenols, antioxidant enzymes (peroxidase, catalase, and polyphenol oxidase) and proline. Fulvic acid (FA), a constituent of soil organic matter, increases the accessibility of nutrients to plants. Biochar (BC) can enhance soil moisture retention, nutrient uptake, and plant productivity during drought stress. That’s why the current study explored the influence of Co, FA and BC on chili plants under drought stress. This study involved 8 treatments, i.e., control, 4 g/L fulvic acid (4FA), 20 mg/L cobalt sulfate (20CoSO_4_), 4FA + 20CoSO_4_, 0.50%MFWBC (0.50 MFWBC), 4FA + 0.50MFWBC, 20CoSO_4_ + 0.50MFWBC, 4FA + 20CoSO_4_ + 0.50MFWBC. Results showed that 4 g/L FA + 20CoSO_4_ with 0.50MFWBC caused an increase in chili plant height (23.29%), plant dry weight (28.85%), fruit length (20.17%), fruit girth (21.41%) and fruit yield (25.13%) compared to control. The effectiveness of 4 g/L FA + 20CoSO_4_ with 0.50MFWBC was also confirmed by a significant increase in total chlorophyll contents, as well as nitrogen (N), phosphorus (P), and potassium (K) in leaves over control. In conclusion4g/L, FA + 20CoSO_4_ with 0.50MFWBC can potentially improve the growth of chili cultivated in drought stress. It is suggested that 4 g/L FA + 20CoSO_4_ with 0.50MFWBC be used to alleviate drought stress in chili plants.

## Introduction

When plants face drought, they exhibit various physiological and biochemical responses^1^. These responses include closing stomata, reducing the rate of water loss through transpiration and lowering the pressure inside plant cells^2^. It also caused membrane damage, induced oxidative stress, disturbed osmotic adjustment, and decreased photosynthetic activity in plants^2–6^. For survival under such stress conditions, plants adopt many strategies, i.e., changes to the structure of the plant, adjustments of osmotic potential in tissue, and strengthening of the antioxidant defences^7^. However, the role of fulvic acid, cobalt, and biochar is vital, and in-depth investigation is still needed to address drought challenges sustainably.

Fulvic acid is an amendment that strengthens plant resilience to drought stress by enhancing nutrient absorption, balancing soil pH, and decreasing fertilizer leaching^8^. It increases aeration, aids in soil water retention by improving the soil's physical and chemical properties and stabilizes soil aggregates. Fulvic acid can also promote microbial activity, which is imperative in nutrient cycling and the availability of essential nutrients to plants^9,10^.

On the other hand, cobalt is a metal that is crucial for producing vitamin B12 in plants^11^. It can help enhance fruit quality, productivity, and plant growth^12^. Decreasing stomatal closure and transpiration rate when applied in growth medium thus improves water usage efficiency in plants. Its supplementation under water stress conditions increases abscisic acid and ethylene levels, which play a key role in regulating the mechanisms that decrease water losses^13^. While elemental cobalt may benefit plants, its limited solubility and availability render cobalt compounds such as cobalt sulfate the preferred choice for agricultural applications and research studies concerning plant nutrition and stress management^11^. Therefore, in our study, we selected cobalt sulfate application.

Biochar sequesters carbon, improves soil properties and enhances water-holding capacity^14^. It also improves ion transfer ability, soil structure, and fertility and reduces heavy metal toxicity. Due to its high porosity, biochar increases microbial activity and nutrient-holding capacity. Research shows that biochar application enhances crop growth, yield, water use efficiency, chlorophyll content, photosynthesis, and leaf water content under drought stress^15,16^. It also strengthens the defense mechanism of plant leaves against drought by enhancing protective enzyme activity and electron transfer.

Chili, scientifically known as *Capsicum annuum L.,* is cultivated worldwide and has significantly increased productivity for spices and vegetables. Chili, an essential ingredient in Pakistani cuisine, is a major exporter, but production has declined due to challenges like drought stress^17^. Originally native to the Americas, it is cultivated in Sindh province, Punjab, and Baluchistan^18^. Chilies exhibit a rich composition characterized by capsaicin, provitamins A, C, and E, and an array of minerals, antioxidants, and secondary metabolites, including carotenoids, phenolic acids, flavonoids, and alkaloids. They were traditionally used as spices, and dry chili fruits have applications in various industries, pharmaceuticals, and cosmetics. However, drought stress significantly deteriorates growth and productivity^19^. Insufficient water availability during critical growth phases negatively affects the progress of plants, leading to smaller yields, reduced productivity, and heightened susceptibility to pests and diseases^17^. Extended periods of drought worsen these difficulties by hindering the plant's ability to produce vital substances like capsaicinoids and vitamins, ultimately compromising the quality and quantity of chili crops^20^.

That’s why, considering the importance of chili, our study focused on applying fulvic acid, cobalt sulfate, and mango fruit waste biochar (MFWBC). We aimed to assess how these substances affect chili growth, chlorophyll levels, antioxidant activities and nutrient concentrations under drought stress conditions. The study addressed the lack of understanding regarding the individual and combined effects of fulvic acid and cobalt sulfate, with and without MFWBC, as soil amendments to alleviate drought stress. It is hypothesized that the combined application of fulvic acid and cobalt sulfate with MFWBC might be a potential amendment for mitigating drought stress in chilies plants.

## Material and methods

### Experimental site

A pot experiment was done in the research area of ResearchSolution (30°15′49″N and 71°30′35″E) to examine the effect of fulvic acid and cobalt sulfate, with and without mango fruit waste biochar (MFWBC), on chili plants cultivated under drought stress. Pre-experimental soil characterization was performed. The characteristics of soil include pHs (8.02)^21^, electrical conductivity [EC*e*] (2.69 dS/m)^22^, organic matter (0.40%)^23^, available phosphorus (4.14 mg/kg)^24^ and extractable potassium (111 mg/kg)^25^.

### Fulvic acid

The CAS Number for fulvic acid was 479–66-3, a product of Cayman Chemicals. It had a molecular formula of C14H12O8, formula weight of 308.2 g/mol, purity ≥ 98%, solid form and originates from a fungus, specifically Penicillium sp. FKP-0046.

### Cobalt sulphate

Cobalt(II) sulfate heptahydrate was purchased from Sigma's certified dealer in Multan. It was identified as Batch Number-0000300865, Product No.-C6768-2.5 KG, MDL No.- MFCD00149657 and CAS No.-10,026–24-1.

### Biochar

The waste of mango fruits was collected from the local fruit market (30°11′30.5"N 71°28′46.9" E). After sun drying, pyrolysis was done under a limited oxygen supply at 500 °C. Finally, grinding was done to pass the biochar from the 2 mm sieve. The characteristics of biochar include: pHs− 7.93, ECe (dS/m) − 4.39, ash content (%)− 40, volatile matter (%)− 20, fixed carbon (%)− 40, total P (%)− 0.99, total N (%)− 0.57 and total K (%)− 1.11.

### Treatment plan and experimental design

The total numbers of treatments were 8, i.e., control, 4 g/L fulvic acid (4FA), 20 mg/L cobalt sulfate (20CoSO_4_), 4FA + 20CoSO_4_, 0.50%MFWBC (0.50 MFWBC), 4FA + 0.50MFWBC, 20CoSO_4_ + 0.50MFWBC, 4FA + 20CoSO_4_ + 0.50MFWBC applied in 4 replicates following completely randomized design (CRD). All the treatments were subjected to drought stress equally.

### Seed collection and sterilization

The chili seeds utilized in the present research came from a licensed seed supplier of the Punjab government in Pakistan. For sterilization of seeds, sodium hypochlorite (5%) was used followed washing with 95% ethanol and deionized water.

### Seeds sowing and thinning

A total of 10 seeds were transplanted on 15 February 2023, with each pot containing 15 kg of soil. The dimensions of the plastic pots were i.e., width = 15 inches and depth = 45 inches. By thinning, 2 healthy seedlings were maintained in each pot.

### Drought

Field capacity, i.e., 40% as drought stress, was maintained using a moisture meter (YIERYI 4 in 1; Shenzhen, Guangdong Province, China) throughout the experiment.

### Fertilizer

To meet the nutritional requirements of chili plants, nitrogen (N) and phosphorus (P) were applied at rates of 25 kg per acre (equivalent to 0.465 g per 15 kg of soil) and 12 kg per acre (equivalent to 0.225 g per 15 kg of soil), respectively. Urea served as the nitrogen source, and single superphosphate provided phosphorus, which was applied at recommended rates. Potassium (K) supplementation was introduced at 12 kg per acre (equivalent to 0.225 g per 15 kg of soil) using potassium sulfate.

### Harvesting and data collection

Plants were collected for data collection after 90 days of transplantation. A meter scale rod was used to measure plant height and fruit length. For fruit yield, weight was taken using analytical balance. However, flexible measuring tape was used for fruit girth.

### Chlorophyll contents

We used the Arnon method to measure chlorophyll* a*, *b*, and total chlorophyll levels in fresh chili leaves^26^. We extracted chlorophyll using 80% acetone and measured absorbance at wavelengths of 663 nm and 645 nm to calculate chlorophyll contents.

### Antioxidants

Superoxide dismutase (SOD) activity was measured using nitro blue tetrazolium (NBT) and taking absorbance at 560 nm^28^. Peroxidase (POD) activity was determined following the procedure described by^29^. The final absorbance was taken at 420 nm. Catalase (CAT) activity was examined by observing H_2_O_2_ decomposition and decrease in absorbance at 240 nm^30^. In ascorbate peroxidase (APX) activity monitoring of ascorbate oxidation was done at 290 nm in presence of H_2_O_2_^31^.

### Malondialdehyde, H_2_O_2_ and proline

Malondialdehyde (MDA) levels were assessed by reacting the sample extract with thiobarbituric acid (TBA) to form a colored complex, and the absorbance of this complex was measured at 532 nm to calculate MDA content^32^. A standard protocol was adopted to analyze free proline in the sample^33^. For the final computation of values, absorbance was taken at 520 nm using a UV–VIS spectrophotometer. Hydrogen peroxide (H_2_O_2_) concentration was determined using a spectrophotometer at 390 nm^34^.

### Electrolyte leakage

Fresh leaf discs of 1 cm diameter were taken in 20 ml of deionized water and incubated at 25 °C for 24 h in test tubes. After incubation, 1st electrical conductivity was quantified^35^

$$\text{Electrolyte Leakage }\left({\%}\right)=\left(\frac{\text{EC}1}{\text{EC}2}\right)\times 100$$

### N, P, K and Na in leaves

To determine leaves phosphorus (P), potassium (K) and sodium (Na) concentration, wet digestion was done using a di-acid mixture, i.e., concentrated nitric and perchloric acid^36^. For nitrogen (N) concentration, analysis sulfuric acid was used^37^. Potassium and sodium were determined via a flame photometer^38^. Phosphorus content was quantified at 420 nm using the yellow color method with a spectrophotometer^38^. The N was assessed following an adapted micro-Kjeldahl procedure^39^.

### Statistical analysis

A standard statistical procedure was followed to analyses the collected data^40^. The collected data was statistically analyzed using the origin software. Tukey test was used for comparison for treatments comparison at *p* < 0.05 ORIGINPRO 2021^41^. Paired comparisons and cluster plots were also made using ORIGINPRO 2021.

### Ethical approval

We all declare that manuscript reporting studies do not involve any human participants, human data, or human tissue. So, it is not applicable. Study protocol must comply with relevant institutional, national, and international guidelines and legislation. Our experiment follows the with relevant institutional, national, and international guidelines and legislation.

## Results

### Plant attributes

**Figure 1:**
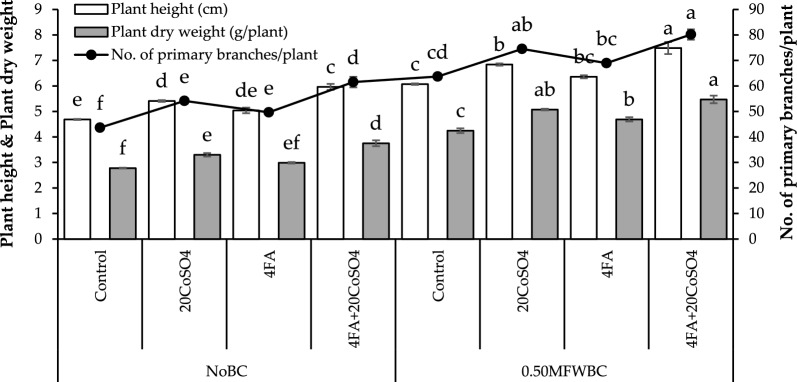
Effect of 4 g/L fulvic acid (4FA) and 20 mg/L cobalt sulfate (20CoSO_4_) with and without mango fruit waste biochar (MFWBC) on plant height, plant dry weight, and no. of primary branches/plant of chili plants. The bars (n = 4 average) with ± S. denoted with distinct letters showed significant changes at *p* ≤ 0.05.

In the no BC, adding 20CoSO_4_ and FA led to a significant increase in plant height over control. The combination of 4FA + 20CoSO_4_ showed a maximum (27.28%) increase in plant height over control. Adding 0.50MFWBC with 20CoSO_4_, FA exhibits an increase in plant height. Applying 0.50MFWBC with 4FA + 20CoSO_4_ showed the highest (23.29%) increase in plant height over the control (Fig. 1).

Plant dry weight increased by adding 20CoSO_4_ and FA under no BC. The combined application of 4FA + 20CoSO_4_ showed the highest (34.94%) rise in plant dry weight over the control. Applying 0.50MFWBC with 20CoSO_4_, FA increased plant height, and their combination 4FA + 20CoSO_4_ showed a maximum (28.85%) increase in plant dry weight (Fig. 1).

Applying 20CoSO_4_, FA, and 4FA + 20CoSO_4_ under no BC increased the no. of primary branches, but the maximum (40.95%) increase was observed with 4FA + 20CoSO_4_ over the control. Adding 20CoSO_4_, FA, and 4FA + 20CoSO_4_ treatments with 0.50MFWBC also shows a significant rise than the control. The highest increase (25.87%) in no. of primary branches was observed with 4FA + 20CoSO_4_ treatment (Fig. 1).

## Fruit attributes

**Figure 2:**
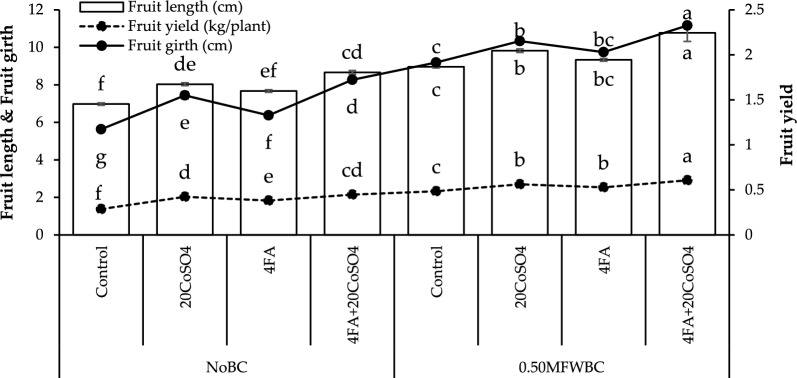
Effect of 4 g/L fulvic acid (4FA) and 20 mg/L cobalt sulfate (20CoSO_4_) with and without mango fruit waste biochar (MFWBC) on fruit length, fruit girth, and fruit yield of chili plants. The bars (n = 4 average) with ± S. denoted with distinct letters showed significant changes at *p* ≤ 0.05.

A significant (24.15%) increase in fruit length was recorded with 4FA + 20CoSO_4_ than 20CoSO_4_ and FA over the control under no BC. Adding 20CoSO_4_ and FA treatments with 0.50MFWBC showed a significant rise in fruit length, but the maximum (20.17%) increase in fruit girth was observed with 4FA + 20CoSO_4_ over control (Fig. 2).

Fruit girth increased by adding 20CoSO_4_ and FA under no BC, but the highest (46.91%) increase was observed with 4FA + 20CoSO_4_ from the control. Applying 0.50MFWBC with 20CoSO_4_, FA increased fruit girth, and their combination 4FA + 20CoSO_4_ showed a maximum (21.41%) increase (Fig. 2).

In the no BC, applying 20CoSO_4_ and FA led to a significant increase in fruit yield than the control. The combination of 4FA + 20CoSO_4_ showed a maximum (55.90%) increase in fruit yield over control. Adding 0.50MFWBC with 20CoSO_4_, FA exhibits an increase in fruit yield. Applying 0.50MFWBC with 4FA + 20CoSO_4_ showed the highest (25.13%) increase in fruit yield over the control (Fig. 2).

## Chlorophyll contents

**Figure 3:**
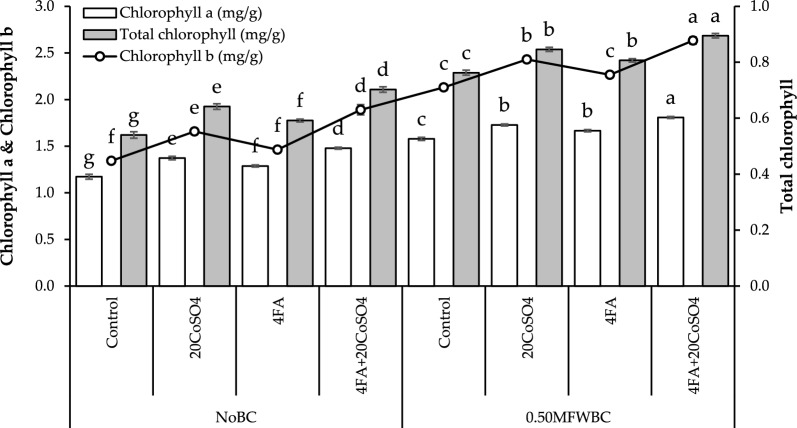
Effect of 4 g/L fulvic acid (4FA) and 20 mg/L cobalt sulfate (20CoSO_4_) with and without mango fruit waste biochar (MFWBC) on chlorophyll* a*, chlorophyll* b*, and total chlorophyll of chili plants. The bars (n = 4 average) with ± S. denoted with distinct letters showed significant changes at *p* ≤ 0.05.

The highest (26.01%) increase in chlorophyll* a* was recorded with 4FA + 20CoSO_4_ than 20CoSO_4_ and FA over the control under no BC. Adding 20CoSO_4_ and FA treatments with 0.50MFWBC showed a significant rise in chlorophyll* a*, but the maximum (14.58%) rise in chlorophyll* a* was observed with 4FA + 20CoSO_4_ over control (Fig. 3).

Chlorophyll* b* increased by adding 20CoSO_4_ and FA under no BC, but the highest (40.78%) increase was observed with 4FA + 20CoSO_4_ from the control. Applying 0.50MFWBC with 20CoSO_4_, FA increased chlorophyll* b*, and their combination 4FA + 20CoSO_4_ showed a maximum (23.59%) increase (Fig. 3).

In the no BC, applying 20CoSO_4_ and FA led to a significant increase in total chlorophyll than the control. The combination of 4FA + 20CoSO_4_ showed a maximum (30.09%) increase in total chlorophyll over control. Adding 0.50MFWBC with 20CoSO_4_, FA exhibits an increase in total chlorophyll. Applying 0.50MFWBC with 4FA + 20CoSO_4_ showed the highest (17.38%) increase in total chlorophyll over the control (Fig. 3).

## Antioxidants

**Figure 4:**
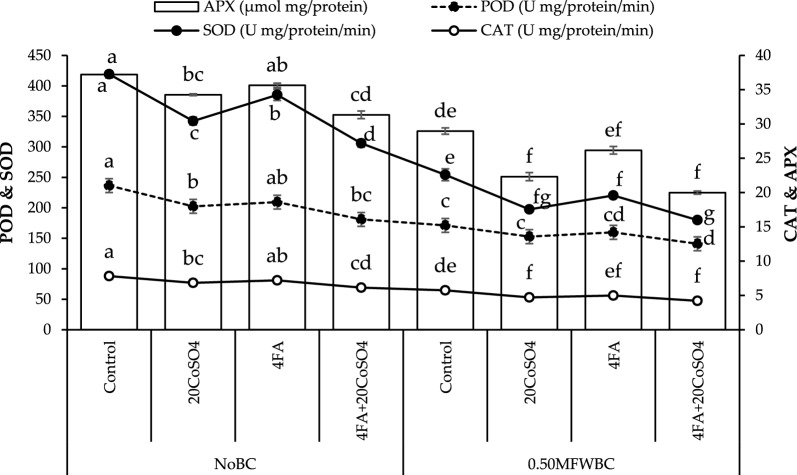
Effect of 4 g/L fulvic acid (4FA) and 20 mg/L cobalt sulfate (20CoSO_4_)with and without mango fruit waste biochar (MFWBC) on peroxidase (POD), superoxide dismutase (SOD), catalase (CAT), and ascorbate peroxidase (APX) of chili plants. The bars (n = 4 average) with ± S. denoted with distinct letters showed significant changes at *p* ≤ 0.05.

Adding 20CoSO_4_ and FA showed a decrease in POD activity under no BC, but the highest (30.64%) decrease was observed with 4FA + 20CoSO_4_ than the control. Applying 0.50MFWBC with 20CoSO_4_, FA exhibited a significant decline in POD activity, and their combination 4FA + 20CoSO_4_ showed the highest (21.32%) decrease (Fig. 4).

SOD activity showed a decrease by the addition of 20CoSO_4_ and FA under no BC, but the highest (37.04%) decrease was observed with 4FA + 20CoSO_4_ than the control. Applying 0.50MFWBC with 20CoSO_4_, FA exhibited a significant decrease in SOD activity, and their combination 4FA + 20CoSO_4_ showed the highest (41.31%) decrease (Fig. 4).

In no BC, applying 20CoSO_4_ and FA led to a significant decrease in CAT activity than the control. The combination of 4FA + 20CoSO_4_ showed a maximum (27.31%) decrease in CAT activity from the control. Adding 0.50MFWBC with 20CoSO_4_, FA exhibits a decrease in CAT activity. Applying 0.50MFWBC with 4FA + 20CoSO_4_ showed the highest (36.21%) decrease in CAT activity over the control (Fig. 4).

In the no BC, applying 20CoSO_4_ and FA led to a significant decrease in APX activity than the control. The combination of 4FA + 20CoSO_4_ showed a maximum (18.73%) decrease in APX activity from the control. Adding 0.50MFWBC with 20CoSO_4_, FA exhibits a decrease in APX activity. Applying 0.50MFWBC with 4FA + 20CoSO_4_ showed the highest (44.85%) decrease in APX activity in comparison to the control (Fig. 4).

## Electrolyte leakage, proline, H_2_O_2_, and MDA

**Figure 5:**
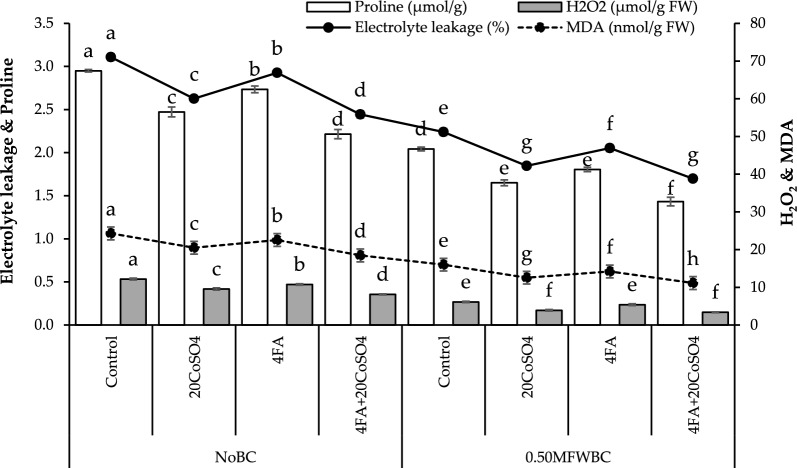
Effect of 4 g/L fulvic acid (4FA) and 20 mg/L cobalt sulfate (20CoSO_4_) with and without mango fruit waste biochar (MFWBC) on electrolyte leakage, proline, hydrogen peroxide (H_2_O_2_), and malondialdehyde (MDA) (D) of chili plants. The bars (n = 4 average) with ± S. denoted with distinct letters showed significant changes at *p* ≤ 0.05.

A more significant (27.24%) decrease in electrolyte leakage was recorded with 4FA + 20CoSO_4_ than 20CoSO_4_ and FA over the control under no BC. Adding 20CoSO_4_ and FA treatments with 0.50MFWBC showed a decrease in electrolyte leakage, but the maximum (31.88%) decrease in electrolyte leakage was recorded with 4FA + 20CoSO_4_ over the control (Fig. 5).

Proline decreased by adding 20CoSO_4_ and FA under no BC, but the highest (33.18%) decrease was observed with 4FA + 20CoSO_4_ from the control. Applying 0.50MFWBC with 20CoSO_4_, FA exhibited a decrease in proline, and their combination 4FA + 20CoSO_4_ showed a maximum (42.58%) decrease (Fig. 5).

In the no BC, applying 20CoSO_4_ and FA led to a significant decrease in H_2_O_2_ than the control. The combination of 4FA + 20CoSO_4_ showed a maximum (50.00%) decrease in H_2_O_2_ from the control. Adding 0.50MFWBC with 20CoSO_4_, FA exhibited a decrease in H_2_O_2_. Applying 0.50MFWBC with 4FA + 20CoSO_4_ showed the highest (81.36%) decrease in H_2_O_2_ over the control (Fig. 5).

A maximum (31.63%) decrease in MDA activity was observed with 4FA + 20CoSO_4_, which was more than 20CoSO_4_ and FA over the control under no BC. Adding 20CoSO_4_ and FA treatments with 0.50MFWBC showed a significant decrease in MDA activity, but the highest (43.75%) decrease in MDA activity was recorded with 4FA + 20CoSO_4_ from the control (Fig. 5).

## Nutrients concentration

**Figure 6:**
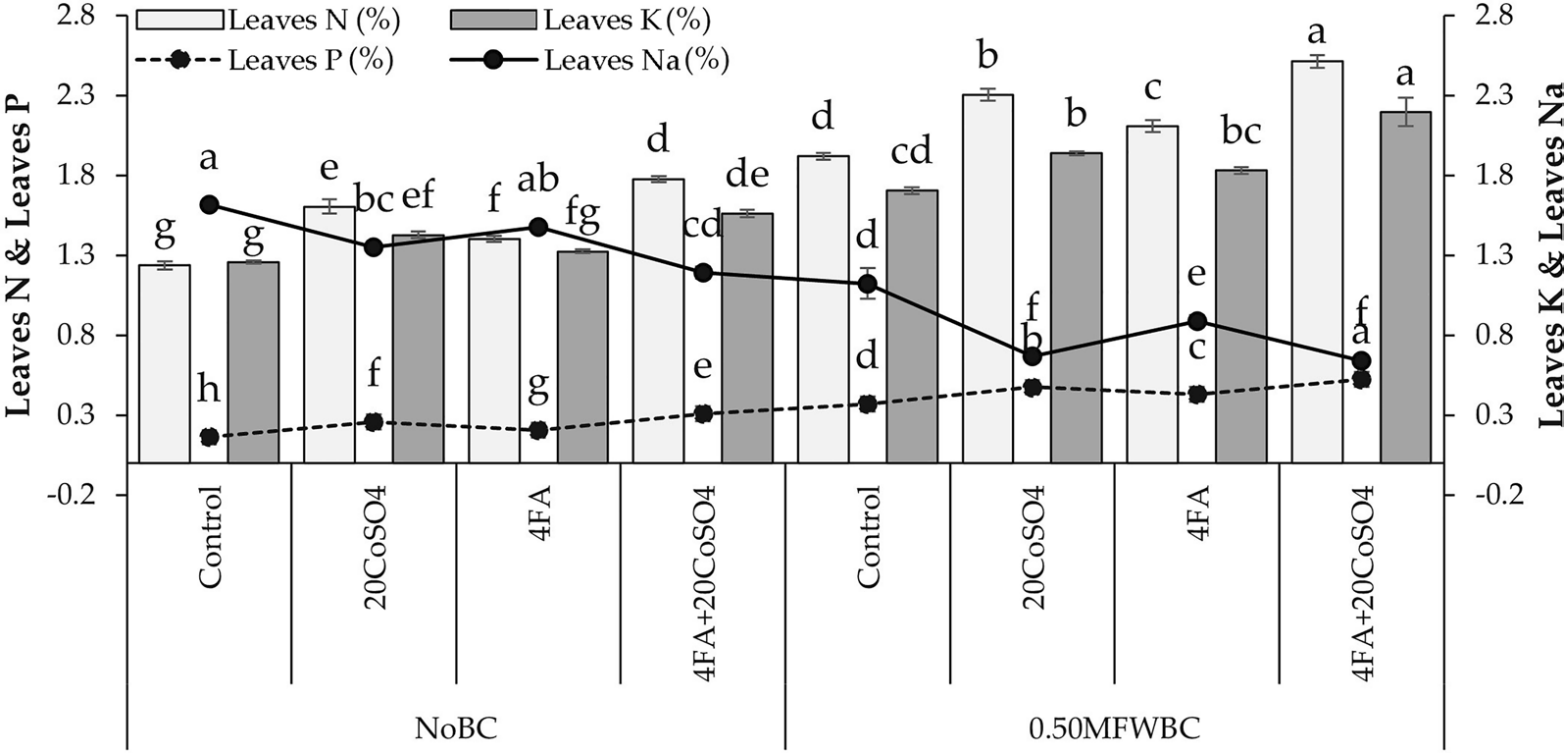
Effect of 4 g/L fulvic acid (4FA) and 20 mg/L cobalt sulfate (20CoSO_4_) with and without mango fruit waste biochar (MFWBC) on leaves N, leaves P, leaves K, and leaves Na of chili plants. The bars (n = 4 average) with ± S. denoted with distinct letters showed significant changes at *p* ≤ 0.05.

The more significant (43.64%) increase in leaves N was recorded with 4FA + 20CoSO_4_ than 20CoSO_4_ and FA over the control under no BC. Adding 20CoSO_4_ and FA treatments with 0.50MFWBC showed a significant rise in leaves N, but the maximum (30.86%) increase in leaves N was observed with 4FA + 20CoSO_4_ over control (Fig. 6).

Leaves P increased by adding 20CoSO_4_ and FA under no BC, but the highest (90.77%) increase was observed with 4FA + 20CoSO_4_ from the control. Applying 0.50MFWBC with 20CoSO_4_, FA exhibited a rise in leaves P, and their combination 4FA + 20CoSO_4_ showed a maximum (41.89%) increase (Fig. 6).

In the no BC, applying 20CoSO_4_ and FA led to a significant increase in leaves K than the control. The combination of 4FA + 20CoSO_4_ showed a maximum (24.06%) increase in leaves K over control. Adding 0.50MFWBC with 20CoSO_4_, FA increased leaves K. Applying 0.50MFWBC with 4FA + 20CoSO_4_ showed the highest (28.74%) increase in leaves K over the control (Fig. 6).

Leaves Na decreased by adding 20CoSO_4_ and FA under no BC, but the highest (35.43%) decrease was observed with 4FA + 20CoSO_4_ from the control. Applying 0.50MFWBC with 20CoSO_4_, FA exhibited a decrease in leaves Na and their combination 4FA + 20CoSO_4_ showed a maximum (59.77%) decrease (Fig. 6).

## Discussion

Drought stress significantly impacts plant growth, particularly during the seedling stage. It leads to a water shortage, causing a chain reaction that impedes development, resulting in plant mortality, decreased pigment content, internal balance disturbance, decreased transpiration, stomata closure, cell shrinkage, and reduced canopy area. Chili seedlings are particularly affected due to the build-up of reactive oxygen species^42,43^. ROS molecules damage essential cellular components and interfere with membrane functions, such as hydrogen peroxide and superoxide ions. The electron transport chain is impacted, lipid peroxidation results, and vital proteins, enzymes, and nucleic acids are rendered inactive by this oxidative stress, which eventually hinders photosynthesis and lowers crop output^44,45^. Drought stress dramatically reduced the growth characteristics, chlorophyll content, fruit yield and development, and nutrient concentrations in the leaves of chili plants.

## Fulvic acid

A naturally occurring substance in soil and organic matter, fulvic acid is essential for promoting root development and facilitating plants' uptake of nutrients and water^8,46^. Fulvic acid attaches itself to nutrients and minerals in the soil to increase its accessibility for plant absorption due to its chelation^47^. Fulvic acid can improve the availability of essential elements, like phosphorus, potassium, and nitrogen, in the soil and promote healthy plant growth^48^.

## Cobalt sulfate

Cobalt sulfate promotes plants in regulating stomatal aperture, activating antioxidant enzymes, enhancing photosynthesis, improving nutrient uptake, and influencing gene expression under drought stress conditions^49^. Cobalt helps by regulating the size of stomata and tiny pores on leaves, which lowers the transpiration rate and aids in water conservation for the plant^50^. Cobalt sulfate stimulates ascorbate peroxidase (APX) and superoxide dismutase (SOD), two essential antioxidant enzymes that are essential for scavenging damaging reactive oxygen species (ROS) generated under drought stress^51^ and protect plant cells from oxidative damage. Cobalt also increases the effectiveness of pigments and enzymes in photosynthesis, allowing chili plants to continue producing energy and essential compounds even under drought stress^52,53^. Lastly, cobalt influences the expression of genes related to drought stress responses, activating mechanisms such as activation of stress-responsive genes, osmotic adjustment pathways, antioxidant defense pathways, cellular signaling pathways, and metabolic pathways that improve the plant's ability to withstand challenging conditions^11,52^.

## Mango fruit waste biochar (MFWBC)

Biochar improves nutrient absorption and regulates antioxidant activity. It has been observed that applying biochar to the soil as an amendment increases the water content of the soil by improving the specific surface area and pore volume^54^. Drought stress affects plants' nitrogen absorption due to soil moisture changes and root function issues, and biochar helps by improving soil water retention and promoting root growth^55,56^. Using biochar reduces oxidative damage to plant cells by activating antioxidant enzymes that scavenge reactive oxygen species (ROS) produced under drought stress^57^. The study found that mango fruit waste biochar improved chili plant photosynthesis and oxidative stress responses under drought stress. It increased leaf water retention capacity, reduced oxidative stress, and reduced malondialdehyde and hydrogen peroxide accumulation, reducing oxidative damage^58^.

## Conclusion

In conclusion, 4 g/L FA + 20CoSO_4_ with 0.50MFWB can potentially increase chili plant growth under drought stress. Treatment 4 g/L FA + 20CoSO_4_ with 0.50MFWB also improves chlorophyll content and nutrients like nitrogen, potassium, and phosphorus, essential for plant growth. Growers are recommended to apply 4 g/L FA + 20CoSO_4_ with 0.50MFWB to mitigate the adverse effect of drought and improve chili plant growth. In future prospects, more investigations are suggested on different crops in variable agroclimatic to declare 4 g/L FA + 20CoSO_4_ with 0.50MFWB as a promising solution for tackling drought stress.

## Data Availability

All data generated or analyzed during this study are included in this published article.
